# The implications of an aging population and increased obesity for knee arthroplasty rates in Sweden: a register-based study

**DOI:** 10.1080/17453674.2020.1816268

**Published:** 2020-09-08

**Authors:** Anders Overgaard, Peder Frederiksen, Lars Erik Kristensen, Otto Robertsson, Annette W-Dahl

**Affiliations:** a The Parker Institute, Copenhagen University Hospital Frederiksberg, Copenhagen, Denmark;; 2> The Swedish Knee Arthroplasty Register, Lund;;; c Faculty of Medicine, Department of Clinical Sciences, Orthopedics, Lund University, Sweden

## Abstract

Background and purpose — Total knee arthroplasty (TKA) has increased substantially in Sweden. We quantified the relative risk for TKA in the Swedish population for different BMI categories and age groups to investigate whether the continued increase in TKA is attributable to increased prevalence of obesity and elderly people in the population, and to put forward model predictions for coming needs for TKA.

Patients and methods — We used the Swedish Nationwide Health Survey (SNHS) and the Swedish Knee Arthroplasty Register (SKAR) 2009–2015 to calculate the relative risk (RR) of TKA by age (middle-aged 45–64 years and elderly 65–84 years) and BMI (BMI 18.5–24.9 normal weight; BMI 25.0–29.9 overweight; BMI > 30 obese). The RR for TKA was applied to the demographic forecasts for the Swedish population as a forecasting model.

Results — Population size increased 5.2% from 2009 to 2015 to 40,000 middle-aged and 250,000 elderly, and the prevalence of obesity increased from 16% to 18% in these 2 age categories. Compared with those of normal weight, the RR for TKA was 2.7 (95% CI 2.5–3.0) higher for the overweight and 7.3 (6.7–8.0) higher for the obese, aged 45–64. The corresponding figures for individuals aged 65–84 were 2.1 (2.0–2.2) and 4.0 (3.8–4.3) higher, respectively. The changes in the prevalence of obesity and an increase in the elderly population accounted for an estimated increase of 1,700 TKAs over the 7 years.

Interpretation — The increase in obesity frequency and the rise in the population of middle-aged and elderly may, to some extent, explain the rise in TKA utilization in Sweden.

A steady increase in knee arthroplasty has been observed (Swedish Knee Arthroplasty Register [SKAR] 2019). The broader acceptance of knee arthroplasty as treatment for knee osteoarthritis (OA) may have led to the rapid increases in utilization and is predicted by some to continue (Kurtz et al. 2007, Kim et al. 2012). Multinational and regional register comparisons show similar tendencies (Robertsson et al. 2010, Kurtz et al. 2011). Different modeling forecasts have attempted to predict future operation needs (Nemes et al. 2014, Inacio et al. 2017) to prepare the healthcare providers.

Obesity is 1 of the major risk factors for development and progression of knee OA that may lead to knee arthroplasty (Felson et al. 1997, Lohmander et al. 2009, Wang et al. 2009). Therefore, as obesity among the population increases, the number of operations is expected to follow a similar pattern. Studies have questioned whether the dramatic rise in operation frequency is attributable to the rise in the prevalence of obesity and the shift in age in the population (Kurtz et al. 2007, Losina et al. 2012, Wills et al. 2012). Following trends and predictions from the United States (Kurtz et al. 2007) where the prevalence of obese individuals in the population has grown at a faster rate than in Sweden, one may speculate that the frequency will continue to increase in Sweden as well.

As the population of the world increases in age and weight the strain on healthcare systems is evident (WHO aging and health[REF?]). Also, the demands for specialized, qualified personnel treating knee OA yield a requirement for studies on the association between demographic changes and the need for arthroplasty surgery to understand and handle coming demands.

We investigated whether the increase in TKA is associated with changes in the prevalence of obesity and the growing elderly population by quantifying the relative risk for TKA in the Swedish population for different BMI categories and age groups.  

## ^Patients and methods^

We used the Swedish Nationwide Health Survey (SNHS) and the Swedish Knee Arthroplasty Register (SKAR) to calculate relative risk (RR) of TKA by age and BMI category. The SNHS comprises a randomly selected national sample of 9,000–10,000 individuals combined with a randomly selected supplementary sample from 4 county councils (Halland, Jönköping, Östergötland, and Kronoberg) and 3 municipalities (Gotland, Göteborg, and Jönköping) consisting of 67,236 individuals (46% men and 54% women) aged 16–84 years. The Sweden Statistics (SCB) provides data on the number of inhabitants divided into different age groups. The SKAR has registered knee arthroplasties since 1975 and has high completeness and correctness of data (SKAR 2019). The response rate for weight and height is > 98% in the SKAR. Weight and height has been validated and was found to have correctness with differences of < 1.5 kg in weight and 1 cm in height between hospital records and what is reported to the SKAR (SKAR 2019). The increase in TKA utilization for different age groups (16–44, 45–64, and 65–84 years) between 2009 and 2015 was estimated by comparing the number of TKAs performed in the period and the operations performed in each BMI category. Increase in population size and prevalence of obesity were calculated similarly. We compared the magnitude of changes in TKA utilization with the changes over the same period regarding population and BMI status. Analyses were performed on the overall population and stratified by age group. We focused on the 2 major age groups who receive TKA (middle-aged 45–64 and elderly 65–84 years), as knee arthroplasty predominates in patients over 45 and under 85 years. To evaluate whether, assuming all other factors remain constant, changes in BMI and population alone could account for the increase in TKA, we combined 2009 with 2015 TKA data with the percentage change in population size, stratified by age group and BMI category. These projections are shown in comparison with the number of TKAs registered in Sweden. Lastly, to assess the future scope of demands on TKAs, we composed a forecast based on current incidence of TKAs (fixed model) and a linear regression model for development in overweight and obesity with population predictions conducted by the SCB.

To calculate the increase in operations for the predicted increase in population the data are extracted from the SCB predicted populations estimate from 2015.

### ^Statistics^

Under the assumption of constant age and BMI surgery rates, we calculated the expected number of operations as the weighted sum of the age and BMI-specific incidence and the corresponding population distribution to illustrate the result of population-composition changes and growth in operation frequency.

$${{}^{\text{n }=}}^{\sum {\text{Rate }}_{\text{age},\text{BMI}}\;\times {\text{ Population }}_{\text{age},\text{BMI}}}$$

Lastly, RR is applied to the demographics of the population ranging from 2005 to 2030 and estimates for the future development of the population from the annual report on development in population done by the SCB. A fixed model was used as static incidents are expected. We used the fixed threshold that was calculated as the average age and BMI-specific incidence rate during the study period. To evaluate our methodology our model predictions are compared with the number of primary TKAs registered in the SKAR.

### ^Ethics, funding, and conflicts of interest^

The data gathering of the Swedish Knee Arthroplasty Register was approved by the Ethics Board of Lund University (LU20-02). The project has been performed in accordance with the Declaration of Helsinki.

The study was supported by a core grant from the Oke Foundation to the Parker Institute. No competing interests were declared.

## ^Results^

### ^Population growth^

The Swedish population increased by 510,335 (5.5%) individuals between 2009 and 2015. In the 7-year period, an increase of 38,638 (1.6%) was seen in the middle-aged (45–64 year) population and an increase of 246,155 (17.1%) in the elderly (65–84 year) population (Table 1).

**Table 1.** Swedish population size and changes in population size by age group, 2009–2015 (SCB)

Factor	2009	2010	2011	2012	2013	2014	2015	Change
Overall	9,340,682	9,415,570	9,482,855	9,555,893	9,644,864	9,747,355	9,851,017	+510,335 (5.5%)
Distribution by age								
45–64	2,407,091	2,412,844	2,418,771	2,425,951	2,429,634	2,434,058	2,445,729	+38,638 (1.6%)
65–84	1,442,601	1,486,029	1,531,341	1,575,315	1,617,178	1,656,400	1,688,756	+246,155 (17.1%)

### ^Increase in obesity^

The prevalence of obesity was relatively static in the period 2009 to 2015 with an increase of less than 3%. When stratifying for age the most relative change in the prevalence of obesity was an increase of 13% (16% to 18%) among both groups of interest (45 to 84 years of age), with a subsequent reduction in the normal-weight population of 9.5% and 7.3% (42% to 38% and 41% to 38%) for the 2 age groups. The change in the number of overweight and obese individuals by an increase in population size between 2009 and 2015 resulted in a relative increase of 14% for overweight and 32% for obese, while the proportion of non-obese individuals decreased by 8% for the middle-aged and increased by 9% for the elderly.

### ^Obesity and age changes and their association with rates of operations^

**Figure 1.**
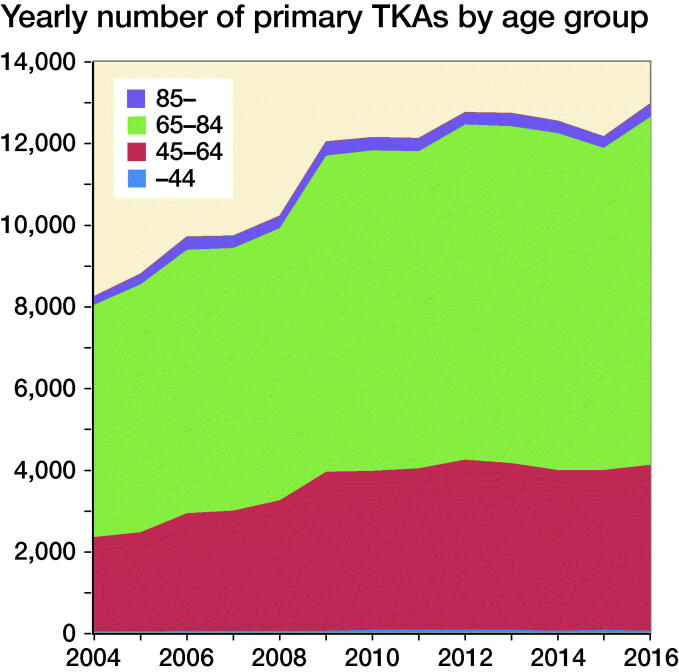
TKAs performed 2004–2016 by age group (SKAR).

**Figure 2.**
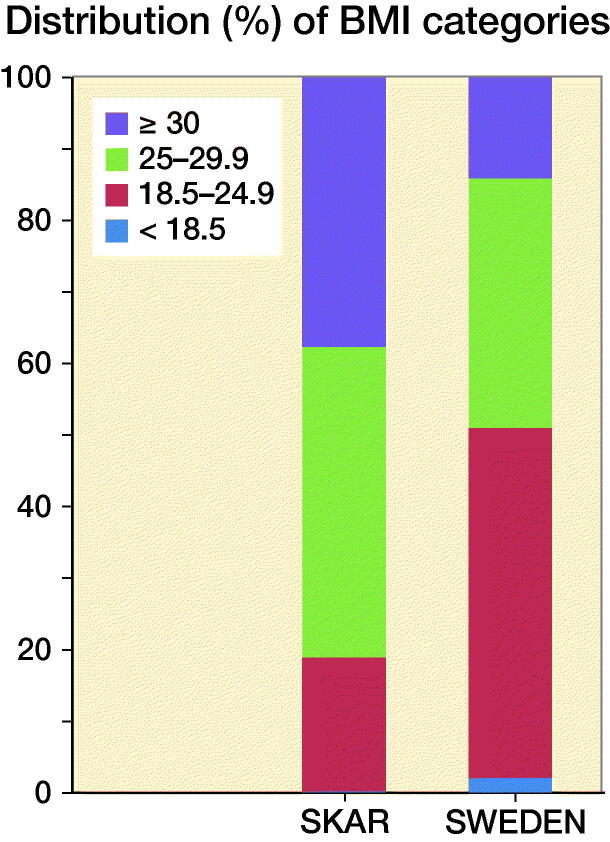
Distribution of BMI categories in the TKA population (SKAR) and the Swedish population (SWEDEN) in 2009–2015.

**Figure 3.**
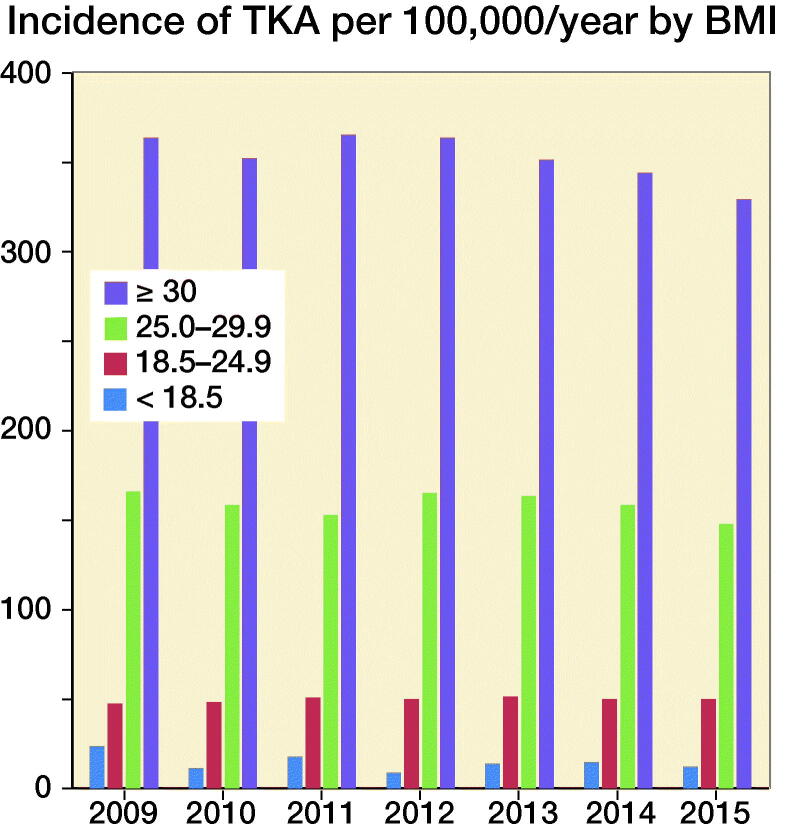
Incidence per 100,000 TKAs 2009–2015 in the different BMI categories.

The annual number of TKAs during 2009–2015 (varying between 12,044 and 12,763), as well as the proportion of patients in the different age groups, was relatively stable (Figure 1). The recipients of TKA compared with the general Swedish population show a higher percentage of overweight (BMI 25–29.9: 44% and 35% respectively) and obese (BMI >30: 38% and 14% respectively) individuals than the Swedish population as a whole (Figure 2). 82% of the TKA population are overweight or obese compared with 49% of the entire Swedish population (Figure 3).

**Figure 4.**
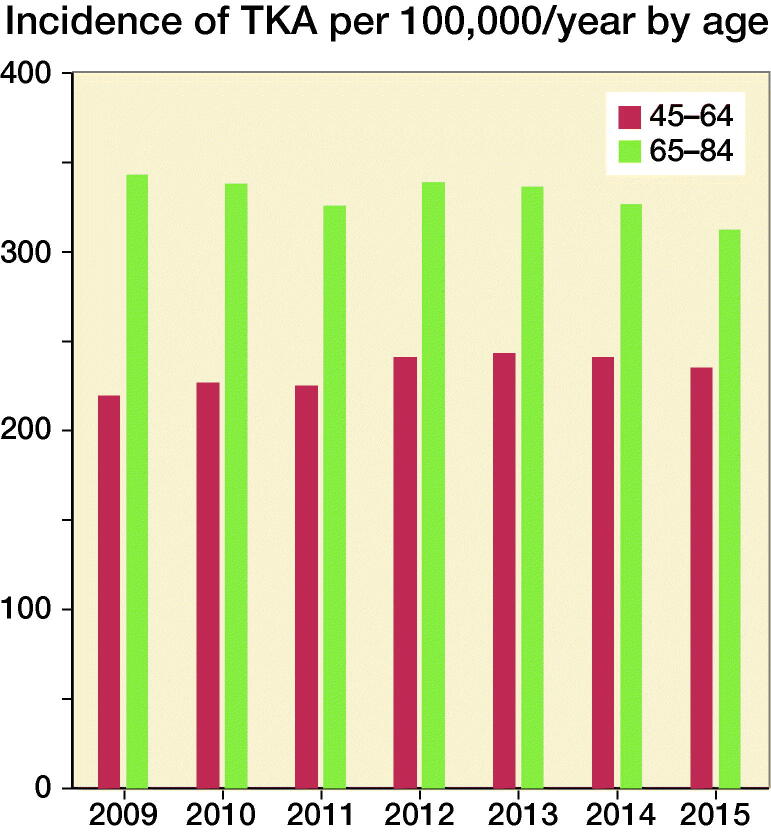
Incidence per 100,000 TKAs 2009–2015 in the different age groups.

The incidence of TKA per 100,000/year in the population of obese elderly shows a slight reduction during the end of the study period (Figures 3 and 4).

The RR for TKA increased with both age and BMI. For the middle-aged patients (aged 45–64), there was a 2.7-fold (95% CI 2.5–3.0) increase with overweight and a 7.3-fold (95% CI 6.7–8.0) higher risk with obesity compared with those of normal weight. Corresponding figures for elderly patients (aged 65–84) were RR 2.1 (95% CI 2.0–2.2) and RR 4.0 (95% CI 3.8–4.3) (Table 2). The growth of the population resulted in an estimated increased need for 1,259 TKAs. Including the increase in obesity in the model resulted in a calculated increase of 1,744 TKAs between 2009 and 2015.

**Table 2.** Calculated relative risk (RR) by age and BMI group compared with normal-weight BMI

Factor	Swedes **^a^**	TKAs **^b^**	Risk/10^5^/year	RR (95% CI)
Age 45–64				
BMI 18.5–24.9	954,746	3,946	59	Reference
BMI 25.0–29.9	1,027,390	11,463	159	2.7 (2.4–2.9)
BMI ≥ 30.0	425,485	12,860	431	7.3 (6.6–8.0)
Age 65–84				
BMI 18.5–24.9	602,479	10,736	251	Reference
BMI 25.0–29.9	662,284	24,725	526	2.1 (2.0–2.2)
BMI ≥ 30.0	265,800	19,136	1,015	4.0 (3.8–4.3)

**^a^** Using the proportion of BMI to calculate the number of Swedes in each group.

**^b^** Total number of total knee arthroplasties in each group 2009–2015.

**Figure 5.**
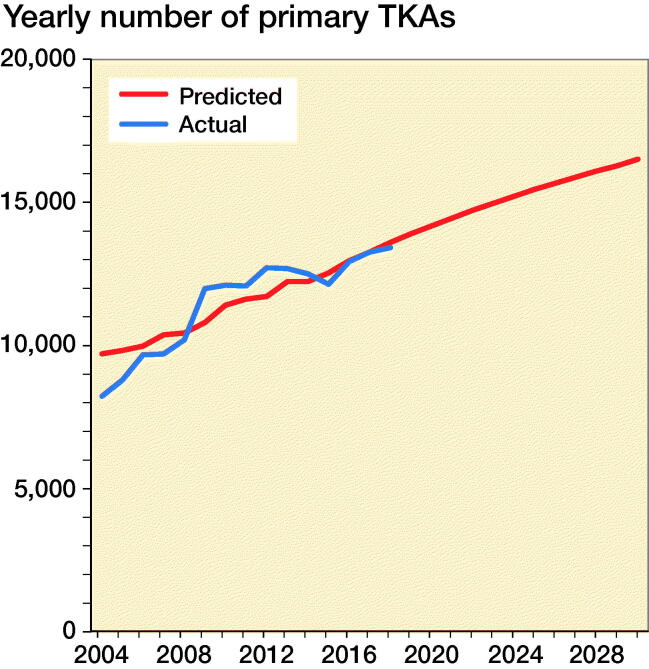
Number of registered and predicted primary TKAs per year 2004–2030.

Applying forecast calculations to the predicted Swedish population increase by the SCB we estimate an increase of primary TKAs for OA to approximately 14,200 operations in 2020 and 16,600 in 2030 (Figure 5).

## ^Discussion^

We found that patients undergoing TKA were more obese than the general Swedish population, and that the magnitude of the Swedish elderly population was increasing at a faster rate than other age groups. Moreover, the increasing BMI in itself was statistically significantly associated with the risk of undergoing knee replacement. The increase in the number of individuals suffering from obesity in the population together with the increase in population size, especially among the elderly, may to some extent explain the continuing increase in TKAs in Sweden. These findings exemplify the growing healthcare costs attributable to obesity and a growing elderly population through increases in operations for OA.

The historical increase in the use of TKA is influenced by a multitude of factors, including a growing prevalence of sports-related knee injuries, a change in physical workloads and a sedentary lifestyle (Felson et al. 1997). Major contributors may also involve the broader acceptance that TKA as a treatment for knee OA has earned among the general population and healthcare providers at large over time. We found a striking association between obesity and risk of surgery. The level of obesity was higher for TKA patients than for the rest of the Swedish population. Further, that increasing obesity as indicated by BMI category was associated with an increased RR for TKA, and that increasing obesity had the most dramatic association with younger patients. For the obese (BMI > 30), the RR for a TKA increased 7-fold compared with the normal weight individual aged 45–64. Using these data we produced a prediction model that may give an explanation for the continued increase in numbers of TKAs performed in Sweden.

If the indications, economic, and TKA use trends remain unchanged, as appears to have been the case over the 7 years, demographic changes in general could explain the increase in TKAs. This seconds the notion that “overweight driven OA” is the major contributor to the rise in operation rates in combination with an increasing elderly population, and that normal or healthy weight may protect against TKAs (Lohmander et al. 2004). This notion is confounded by the use of TKA as a surrogate endpoint for late-stage OA in the population. Though there is a shared pathogenetic phenotype of OA and obesity the frequency of OA in the population is hard to quantify. In fact, only for a lesser subgroup of patients suffering from OA who need an arthroplasty might obesity be the deciding factor driving the OA patients to surgery, as obesity alone affects locomotion and self-rated health (Marks 2007, Silverwood et al. 2015).

Kurtz et al. (2007 revised 2014) calculated an increase of over 600% TKA operations in the United States from 2005 to 2030 based on trajectories from the rapid increases seen between 1990 and 2003, when the increases in numbers of arthroplasty performed increased rapidly. This model is not applicable to the Swedish population, where trends of incidences have slowed. Cross-sectional studies from this period (Kurtz et al. 2007, Losina et al. 2012) show that obesity and demographic changes cannot explain the full extent of the explosive TKA increase in the United States and Germany. Increases in use in younger patients due to sports-related injuries and direct-to-consumer advertisements may influence the public awareness of treatment options as described by Dieppe et al. (1999) for publications from 1990–1998 with considerable variance in the use and demand across nations and healthcare models. A more conservative estimate may be warranted in the Scandinavian countries where the incidence historically is lower. More recent calculations expecting continued explosive growth is rejected and logarithmic development to Poisson’s models describes the differences in mathematical approaches (Sasieni and Adams 1999, Inacio et al. 2017). The main criticism of such data-driven models as presented by Culliford et al. (2015) is that they do not involve any estimate for planning and supply-side constraints (capacity of surgeons and hospital units) and restrictions, as is the case in this model. Regardless of modeling attempts, aging and increasing BMI is part of what is driving the TKA patient increase. And mathematical modeling per se is an attempt to “fit” the trends expressed in the registries. We do not expect current limitations in specialized personnel in Sweden to be an issue. However, for the Nordic countries use of TKA seems to have reached a plateau, therefore modeling expecting continued explosive growth as in the United States is rejected. The fact that TKA use in Sweden follows the needs and demands from the public and lawmakers is evident in upholding the waiting-list guarantees of no longer than 90 days wait for surgery (before COVID-19). Though there are strengths in the data sources, our analyses are limited by the fact that population, obesity, and TKA data were each obtained from different sources, limiting the ability to compare in-group changes. Additionally, we did not have the possibility to analyze supply-chain limitations and therefore our estimate may only be indicative for the immediate future TKA frequencies. The SKAR has collected data on BMI since 2009, thus limiting the opportunity to compare earlier more marked changes in operation rates. Prior to 2009 more dramatic changes in the number of operations were seen and might to an extent be influenced by the factors mentioned above. This deems the data unfit for a fixed calculation model as BMI-defined incidence would follow trends of usage and therefore would not explain their contribution. Nationwide register-based studies like the present one have the apparent strength of being population-based, thus reducing the risk of selection bias. However, some degree of residual confounding, bias, and misclassification cannot be ruled out.

For the Swedish population and Swedish healthcare providers, assuming unchanged indications and utilization patterns, we can evaluate the consequence of demographic changes and changes in overweight and obesity in the population. The reliability of the forecast model diminishes as new prosthesis types and centralization of treatment and political intervention as “treatment guarantees” change the way patients are treated over time; though stable at the moment, although the increase in obesity in Sweden in a longer perspective than 2009–2015 is relatively “large,” for the studied period increases were meager. These observations on RR highlight the need for information on BMI if one is to compare and combine data from registers as this is shown to have large implications for the incidence of operations. The increase in obesity is likely to be a contributor to the increases seen in national registers globally.

The lack of medical treatment options for OA leaves patients seeking surgery, and preventing or postponing surgery by treatment of modifiable risk factors like obesity should be a key objective for halting increasing numbers of TKAs being performed. The attributable fraction among exposed knee OA patients with concomitant overweight or obesity is confounded by the inherent limitations that patients with knee OA and overweight/obesity suffer from the consequent additional problem constituted by difficulties in exercising.

In conclusion, we have shown a striking association of obesity and an increasingly elderly population with the rise in TKA rates. Most likely, the continuous increase in, and aging of, the Swedish population will lead to a higher demand for intervention for OA and an increased burden on healthcare resources and hospital budgets.
